# Protocol for a Systematic Review and Meta-Analysis of Lithium, Anticonvulsive or atypical antipsychotic Drugs for Treatment of Refractory Obsessive-Compulsive Disorder

**Published:** 2017

**Authors:** R Soleimani, MM Jalali, A Keshtkar, SM Jalali

**Affiliations:** * Psychiatric Department, Shafa Hospital, Guilan University of Medical Sciences, Iran; ** Otolaryngology Department, Amiralmomenin Hospital, Guilan University of Medical Sciences, Iran; *** Endocrinology & Metabolism Research Institute, Tehran University of Medical Sciences, Iran; **** Pharmacy Faculty, Mashhad University of Medical Sciences, Iran

**Keywords:** obsessive-compulsive, neuropsychiatric, lithium, anticonvulsive agent, physiopathology, antipsychotic drug

## Abstract

Introduction. Obsessive-compulsive disorder (OCD) is a neuropsychiatric disorder that causes significant distress to the afflicted individual. About half of OCD patients treated with an adequate trial of serotonin reuptake inhibitors fail to fully respond to treatment and continue to exhibit significant symptoms. Therefore, there is a need for other agents to alleviate the symptoms of these disorders. In spite of considerable research including numerous randomized controlled trials and systematic reviews, there exists uncertainty regarding what treatments are effective. In this systematic review, we evaluated the efficacy of mood stabilizers in treatment-refractory OCD.

Materials and methods. We conducted a meta-analysis of all randomized clinical trials evaluating lithium, anticonvulsive agents or atypical antipsychotic drugs for OCD to determine which therapies show more effective than a placebo, in reducing obsessive-compulsive symptoms. We acquired eligible studies through a systematic search of Cochrane Central Registry of Controlled Trials, MEDLINE, EMBASE, PsycINFO, Scopus, ProQuest and Google scholar.

We conducted meta-analyses to establish the effect of lithium, anticonvulsive agents, or atypical antipsychotic drugs on patient-important outcomes when possible. To assess relative effects of treatments, we constructed a random effect model.

Discussions. Our review was the first to evaluate all treatments for OCD, to provide the relative effectiveness of lithium, anticonvulsive agents, or atypical antipsychotic drugs, and prioritize patient-important outcomes with a focus on functional gains. Our review facilitated the evidence-based management of patients with resistant OCD, and identified the key areas for future research.

## Background

Obsessive compulsive disorder (OCD) is a psychiatric disorder which has an estimated 12-month prevalence of 1.2 percent and an estimated lifetime prevalence of 2.3 percent [**1**][**2**]. Other authors have reported a prevalence between 2% and 3.5%, without indicating specific countries [**3**]. OCD could be started as early onset (before age 25) or late onset, persists throughout a person’s life, and produces a substantial impairment in functioning due to the severe and chronic nature of the illness [**4**]. Patients with OCD have a poorer overall quality of life, and experience significant impairment in academic functioning, work performance and interpersonal relationships [**5**][**6**][**7**]. 

The Diagnostic and Statistical Manual of Mental Disorders, Fifth Edition (DSM-5), released in 2013, includes a new chapter for OCD and related disorders, including body dysmorphic disorder, hoarding disorder, trichotillomania, and excoriation disorder. The criteria for the disorder include two components: obsessions and compulsions. Obsessions are recurrent and persistent thoughts, urges, or images that are experienced, at some time during the disturbance, as intrusive and unwanted, and that in most individuals cause marked anxiety or distress. The individual attempts to ignore or suppress such thoughts, urges, or images, or to neutralize them with some other thought or action (i.e., by performing a compulsion). Compulsions are repetitive behaviors or mental acts that the individual feels driven to perform in response to an obsession, or according to rules that must be applied rigidly. The behaviors or mental acts are aimed at preventing or reducing anxiety or distress or preventing some dreaded event or situation; however, these behaviors or mental acts either are not connected in a realistic way with what they are designed to neutralize or prevent, or are undeniably excessive [**4**].

Treatment-resistant OCD patients are described as those who received adequate trials of first-line therapies, but a reduction in their Yale-Brown Obsessive-Compulsive Scale (Y-BOCS) is <25% or <35% with respect to baseline [**8**]. An adequate trial of first-line therapies is described as at least 10-12 weeks of the highest tolerated dose of serotonin reuptake inhibitors [**9**]. In another description, Pallanti and Quercioli defined treatment response stages; 35% or greater reduction in Y-BOCS as “full response,” >=25% but <35% as “partial response,” and <25% as “nonresponse” [**10**]. 

This systematic review examines the efficacy and safety of lithium, anticonvulsant agents, or atypical antipsychotic drugs as an augmentation strategy for treatment-refractory OCD in recently conducted, double-blind, randomized control clinical trials. Numerous psychiatric disorders co-occur in people with OCD at rates higher than in the general population, including major depressive disorder and other anxiety disorders, but, in this review, we focused exclusively on trials in participants with a primary diagnosis of OCD.

## Description of the intervention

Although Serotonin (5-hydroxytryptamine 5-HT) reuptake inhibitors, especially selective 5-HT reuptake inhibitors (SSRIs), and clomipramine are recommended as first-line agents for drug treatment of OCD [**11**], it is estimated that more than 40-60% of the SSRIs treated population are treatment-resistant [**12**] and an even higher proportion of patients fail to experience complete remission of their symptoms [**13**]. Therefore, there is a need for other agents to alleviate the symptoms of these disorders.

There are lots of strategies to augment the treatment response in nonresponsive patients ranging from adding cognitive-behavioral therapy to pharmacotherapy augmentations such as antipsychotics, a combination of another serotonin reuptake inhibitors, anticonvulsants, or even the use of neurosurgical procedures [**9**].

Notwithstanding, the emergence or exacerbation of obsessive–compulsive symptoms in patients with a primary diagnosis of psychosis treated with atypical antipsychotics has been described [**14**][**15**].

In the case of non-response, a change to another SSRI is suggested [**16**][**17**]. Because of the high number of OCD subjects not responding sufficiently even to a switch [**18**], the evaluation of additive therapeutic options has high clinical relevance. Two pharmacological augmentation strategies have been implemented to aid patients non-responding to SSRI monotherapy. The first category of augmentation strategies involves the use of serotonin-enhancing agents (such as lithium, clonazepam, and buspirone) to maximize treatment response. The second category of augmentation strategies has involved the addition of low-dose dopamine antagonists to SSRI medications.

Drugs commonly classed as mood stabilizers include lithium, anticonvulsants (such as valproic acid, lamotrigine, carbamazepine, oxcarbazepine), and atypical antipsychotics. There is insufficient evidence to support the use of mood stabilizers in the treatment response of OCD patients.

## How the intervention might work

The etiology of OCD is unclear. While it is widely accepted that serotonergic mechanisms are important in the neurobiology of OCD [**19**], other neurotransmitter systems may also be involved. GABA is the principal inhibitory neurotransmitter in the brain, and it may also play a role in cortical disinhibition [**20**]. Observations of the potential anxiolytic effect of the anticonvulsant topiramate suggest a role for GABA neurotransmission in OCD. Topiramate was shown to alleviate obsessive behavior in two OCD studies [**21**][**22**]. 

Dysfunction in cortico–thalamic–striatal circuits is an integral component of OCD-like behavior. The neuroimaging studies in OCD patients showed that the orbitofrontal cortex, cingulate cortex, basal ganglia and parietal cortex had been implicated in the pathophysiology of OCD [**23**][**24**][**25**]. It is hypothesized that dysfunction in neurotransmitter systems like the serotonergic, dopaminergic, and glutamatergic systems is implicated in OCD pathology [**26**].

Mood stabilizers are frequently used to treat bipolar disorder, schizophrenia, and obsessive–compulsive behavior in Huntington’s disease. Carbamazepine and lamotrigine primarily block neuronal sodium channels by binding to its common recognition site [**27**][**28**]. Carbamazepine also inhibits calcium channels in rat hippocampal neurons [**29**]. Although it is not clear whether the inhibition of these channels by carbamazepine and lamotrigine is linked to psychotropic activity, these inhibitory effects are closely related to the antiepileptic effects. The principal pharmacological effect of valproate is thought to be an increase in GABA transmission [**30**]. Sodium valproate increases GABA synthesis and release in some specific brain regions such as substantia nigra [**31**]. Lamotrigine also increases GABA release but decreases glutamate release in the rat entorhinal cortex [**32**]. These enhancing effects on the GABA transmission might be involved in the psychotropic activity. Furthermore, mood stabilizers exert a certain influence on dopamine and 5-HTtransmission. These findings suggest that these enhancing actions on the dopamine and 5-HT transmission in the striatum, prefrontal cortex, and hippocampus could be correlated with the psychotropic activity. On the other hand, lithium decreases dopamine release in the nucleus accumbens, and it did not affect the prefrontal cortex [**33**]. Therefore, the relevant targets of lithium’s action are considered to be inositol depletion and glycogen synthase kinase-3 (GSK-3) inhibition [**34**].

Prepulse inhibition (PPI) is defined as a reduction in the startle reflex elicited by weak sensory prestimulation, and it is thought to reflect the operation of a sensorimotor gating system in the brain. Impaired PPI has been reported in patients with OCD [**35**][**36**]. Mood stabilizers potentiate the PPI [**26**].

## Why it is important to do this review

Given the frequent off-label use in practice, the uncertain efficacy, the side effects, and the high costs of these drugs in OCD, a systematic review is important. Also, the pharmaceutical industries attempt to find other indications for their compounds. Therefore, there is a need for an up-to-date systematic review. The latest available meta-analyses evaluating atypical antipsychotic augmentation therapies in treatment-resistant OCD are based on databases up to December 2013 [**37**]. 

## Objectives

Our question for the systematic review is “For adults who have OCD which has failed to respond to at least one trial of a serotonergic reuptake inhibitor, will lithium, an anticonvulsive agent or an atypical antipsychotic drug be more effective than a placebo, in reducing obsessive-compulsive symptoms?” 

Our secondary aims are to determine: (a) if the proportion of treatment responders and mean difference in Y-BOCS ratings are influenced by length of time the therapy with anticonvulsive augmentation is followed and (b) the side effects of lithium, anticonvulsive agents or atypical antipsychotic drugs compared to placebo when added as an augmentation agent to SSRI monotherapy in treatment-resistant OCD patients.

## Methods

**Criteria for considering studies for this review**

**Types of studies**

All clinical trials with control group will be included. Randomized cross-over trials will be eligible, but only data up to the point of first cross-over will be used because of the instability of the problem behaviors and the likely carryover effects of the treatments. We also will include cluster-randomized trials that meet certain criteria. Case studies and case reports will be excluded.

**Types of participants**

Trials will be included if

1 They described adults (aged 18 years or older) who had a diagnosis of OCD according to the DSM-III/DSM-IV/DSM-V (300.3) [4,38-41] or ICD-10 (F 42) [42].

2 They used the Yale-Brown Obsessive Compulsive Scale (Y-BOCS) [43] as a primary outcome measure. The Y-BOCS is a 10-item clinician-rated scale, which is widely used to measure the severity of obsessive-compulsive symptoms, which has a total score range of 0 to 40. Higher scores indicate greater symptomatology of OCD.

3 Participants had persistent symptoms of OCD defined as a Y-BOCS score of 16 or more.

4 Participants had had at least one adequate trial of a SSRI or clomipramine. An adequate trial of a SSRI or clomipramine was defined as a maximum dose tolerated for at least 8 weeks prior to randomization.

5 Participants remained on the SSRI or clomipramine for the duration of the trial.

6 The study compared lithium or anticonvulsive agents or atypical antipsychotic drugs and placebo augmentation.

7 They had a trial end point of at least 4 weeks.

We only included systematic reviews evaluating children and adults if separate results were available for adults. We excluded trials in participants with a primary or secondary diagnosis of another axis I or axis II disorder if these made up for more than 20% of the participants. We did not exclude any OCD trials in participants with a serious concomitant medical illness.

**Types of interventions**

The intervention had to be lithium or antiepileptic medications or atypical antipsychotic drugs that are mentioned in the British National Formulary 61 (BNF March2011) [**44**]. There were no limits in terms of study duration.

1. Experimental treatments included lithium or one of the following anticonvulsive agents: carbamazepine, eslicarbazepine, ethosuximide, lamotrigine, oxcarbazepine, phenobarbital, primidone, phenytoin, rufinamide, topiramate, valproate and vigabatrin or one of the following atypical antipsychotic drugs: olanzapine, risperidone, quetiapine, ziprasidone, aripiprazole, paliperidone, asenapine, lurasidone, cariprazine.

2. Comparator substances were either placebo.

3. Treatments have been given as augmentation therapy. We excluded trials with only non-pharmacological treatments as a comparator. There were no limits in terms of duration of treatment.

**Types of outcome measures**

**Primary outcomes**

Our primary outcome measure was the proportion of treatment responders (as defined by a 35% decline in Y-BOCS scores) in the augmentation group compared to the placebo group. A 35% decline in Y-BOCS rating was chosen as the threshold for treatment response based on the definition of full treatment response suggested by the International Treatment Refractory OCD Consortium [**45**].

**Secondary outcomes**

1. Yale-Brown Obsessive Compulsive Scale (Y-BOCS) scores at the end of the trials.

2. Premature trial discontinuation due to any reason, to the inefficacy of treatment, or to adverse events.

3. Adverse events (such as sedation or extrapyramidal side effects).

We classified the outcomes as short-term (up to six months), medium-term (seven to 12 months) and long-term (longer than 12 months).

**Search methods for identification of studies**

**Electronic searches**

We will search the following sources from inception to the present.

• The Cochrane Central Register of Controlled Trials (CENTRAL)

• MEDLINE (via Pubmed, from 1950 to the present)

• EMBASE (via Scopus, from 1980 to the present)

• PsycINFO

• Scopus

• ProQuest

• Google scholar

For detailed search strategies, see **Table 1**. We used PubMed’s ’My NCBI’ (National Center for Biotechnology information) email alert service for the identification of newly published systematic reviews using a basic search strategy (see Appendix 1).

**Table 1:** Search strategies

The search terms used	
#1	obsess* OR compul* OR OCD
#2	lithium OR anticonvuls* OR antiepileptic OR antipsychotic
#3	carbamazepine OR Tegretol OR Carbazepin OR Epitol OR Finlepsin OR Neurotol OR Amizepine
#4	eslicarbazepine
#5	ethosuximide OR Ethosuccimid OR Ethylmethylsuccimide OR Ethymal OR Zarontin OR Petnidan OR Pyknolepsinum OR Suksilep OR Suxilep OR Emeside
#6	lamotrigine OR Crisomet OR Lamictal OR Lamiktal OR Labileno
#7	oxcarbazepine OR Timox OR Trileptal
#8	phenobarbital OR Phenobarbitone OR Phenylethylbarbituric OR Phenemal OR Phenylbarbital OR Hysteps OR Luminal OR Gardenal
#9	primidone OR Misodine OR Desoxyphenobarbital OR Resimatil OR Sertan OR Mysoline OR Mylepsinum OR Apo-Primidone OR Primaclone OR Liskantin OR Mizodin
#10	phenytoin OR Diphenylhydantoin OR Fenitoin OR Diphenylhydantoin* OR Difenin OR Dihydan OR Epamin OR Epanutin OR Hydantol OR Antisacer OR Dilantin
#11	rufinamide OR Inovelon
#12	topiramate ORTopamax OR Epitomax
#13	valproate OR Valproic OR Divalproex OR Propylisopropylacetic OR Propylpentanoic OR Convulsofin OR Depakene OR Depakine OR Depakote OR Vupral OR Divalproex OR Ergenyl OR Dipropyl Acetate
#14	vigabatrin OR gamma-Vinyl-GABA OR (gamma Vinyl GABA) OR (gamma-Vinyl-gamma-Aminobutyric Acid) OR Sabril*
#15	olanzapine OR risperidone OR quetiapine OR ziprasidone OR aripiprazole OR paliperidone OR asenapine OR lurasidone OR cariprazine
#16	#3 OR #4 OR #5 OR #6 OR #7 OR #8 OR #9 OR #10 OR #11 OR #12 OR #13 OR #14 OR #15
#17	#2 OR #16
#18	#1 AND #17

If we detected additional relevant key words during any of the electronic or other searches, we modified the electronic search strategies to incorporate these terms and document the changes. We placed no restrictions on the language of publication when searching the electronic databases or reviewing reference lists in identified studies.

**Fig. 1:**
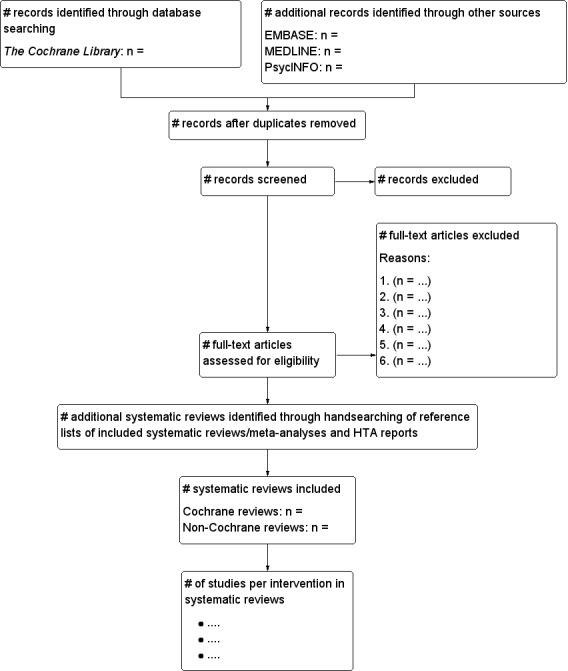
Systematic review selection flowchart

We tried to identify other potentially eligible systematic reviews by searching the reference lists of retrieved systematic reviews, meta-analyses, and health technology assessment reports. We presented the results of the screening in a flowchart (**Fig. 1**).

**Ongoing Trials databases**

We searched the following ongoing trials registers to identify relevant trials:

The metaRegister of Controlled Trials on www.controlled-trials.com.

The US National Institutes of Health Ongoing Trials Register on www.clinicaltrials.gov.

The World Health Organization International Clinical Trials Registry Platform on www.who.int/trialsearch.

**Searching other resources**

**Handsearches**

Handsearches were not done for any specific journals.

We scanned the bibliographies of the included studies, relevant reviews, government reports and other “grey literature” for relevant references, a process referred to as “snowballing”.

**Grey literature and request for information**

Grey literature refers to reports that are difficult to find via conventional channels such as published journals. Examples of grey literature include technical reports from government agencies or scientific research groups, working papers from research groups or committees, white papers, conference proceedings and abstracts, theses and dissertations, or unpublished research reports.

We inspected the references of all identified trials and previous reviews for more trials. We contacted the first author of each included trial for missing information and the existence of further trials. We contacted the manufacturers of lithium and anticonvulsive drugs and asked them about further relevant trials and for missing information on identified trials.

**Data collection and analysis**

**Selection of studies**

Firstly, one reviewer evaluated the titles and abstracts to determine whether the study met the eligibility criteria. Secondly, full texts were assessed independently by two reviewers (S.R., J.M.M.), for eligibility. Disagreements were resolved by a discussion to reach consensus.

**Data extraction and management**

Two review authors (S.R., J.M.M.) independently extracted the data from the full texts of included studies using a specifically developed extraction form. The data extraction form has been piloted previously.

Information was collected on the following:

1. Study characteristics (first author, geographical origin, year of publication, start and end of study, study design, number of arms, sample size, duration of follow-up).

2. Participant characteristics (age, sex, number of participants, how diagnosis was performed, case definitions, disease manifestations, inclusion and exclusion criteria in the included studies, baseline imbalances between study arms and possible confounders (disease manifestation, delay between onset of symptoms and treatment, previous treatment, co-medication, co-morbidities and other confounders as reported by the authors).

3. Intervention and comparator details (sample size for each treatment arm, blinding, dose and type of interventions, dosage adjustment based on body weight, duration of treatment, withdrawals and dropouts).

4. Outcome measures (description of measurement tools used, data for continuous/ dichotomous/ categorical efficacy variables, the time point of measurement, adverse events, and serious adverse events).

When adjusted analyses were available in primary studies, these adjusted estimates of treatment effects were used. Otherwise, we extracted the unadjusted data as reported in the primary study. This fact was considered accordingly in the risk of bias assessment and was subject to sensitivity analyses. Data was entered into Review Manager (RevMan 5.3) by one of the reviewers and checked by a second reviewer. Discrepancies in data extraction or entry were resolved by a discussion to reach consensus. Reviewers were not blinded to study author, journal, or institution.

**Assessment of methodological quality of included studies**

The assessment of risk of bias was performed by two reviewers, independently, considering the following domains according to the Cochrane risk of bias tool: sequence generation, allocation concealment, blinding (of participants, personnel, and outcome assessors), incomplete outcome data, selective outcome reporting, and other sources of bias for the RCTs. According to the Cochrane Handbook, these items will be described as having a ‘low’, ‘high’, or ‘unclear’ risk of bias [**46**].

**Measures of treatment effect**

1. Binary data

We calculated the odds ratio (OR) and its 95% confidence interval (CI).

2. Continuous data

We calculated the mean differences as it preserved the original units and was, therefore, easier to interpret.

2.1 Change versus endpoint data

We used change data only when endpoint data were not available.

2.2 Skewed data

Continuous data on clinical and social outcomes were often not normally distributed. To avoid the pitfall of applying parametric tests to non-parametric data, we applied the following standards to all data before inclusion: 

(a) Data from trials of, for example, at least 200 participants were entered in the analysis irrespective of the following rules because skewed data pose less of a problem in large trials.

(b) Endpoint data: when a scale started from the finite number zero, we subtracted the lowest possible value from the mean, and divided this by the standard deviation. If this value was lower than one, it strongly suggested a skew and we excluded the trial. If this ratio was higher than one but below two, there was a suggestion of skew. We entered the trial and test whether its inclusion or exclusion changed the results substantially. If the ratio was larger than two, we included the trial, because skew was less likely [**46**][**47**].

(c) When continuous data were presented on a scale which included a possibility of negative values (such as change data), it was difficult to tell whether data were skewed or not. We entered the trial, because change data tended to be less skewed and because excluding trials also led to bias, as not all the available information have been used.

**Unit of analysis**

The unit of analysis was each patient recruited in the studies.

**Dealing with missing data**

Data were analyzed on an intention-to-treat basis whenever possible. If data were only available in graphical format, we thoroughly estimated the numerical values.

**Assessment of heterogeneity**

Heterogeneity among studies was investigated by using the chi2 test and I2 test. If significant heterogeneity was detected (I2 > 50% or p <0.1) for outcome measures, the calculations with a fixed effect model was repeated using a random effects model as sensitivity analysis, and we considered results from both.

**Assessment of reporting biases **

We planned to minimize the impact of reporting bias in our systematic review by ensuring a comprehensive search for eligible studies including three trial registries. A funnel plot and appropriate statistical tests for small study effects were performed if ≥10 studies were available [**48**].

**Data synthesis **

Intervention effects in divergent study designs were influenced differently by bias. Estimation of treatment effects were based on a fixed effect model; when we were faced with substantial heterogeneity (i.e., I2> 50%), a random effects model was calculated as well as a sensitivity analysis. We calculated pooled RRs and 95% CIs across comparable studies using Review Manager (RevMan 5.3). When considerable heterogeneity (I2 > 80%) was found between comparable studies, pooled estimates were not provided. Instead, a descriptive synthesis of findings was performed.

**Subgroup analysis and investigation of heterogeneity**

Due to the clinical importance of treatment options, subgroup analyses focusing on different agents were of considerable interest. We evaluated prespecified classes of drugs (lithium or anticonvulsive drugs) in subgroup analyses.

**Sensitivity analysis**

We planned to test the robustness of the results by repeating the analysis using a random effects model when confronted with substantial heterogeneity.

## Discussion

Controversy exists about the choice of drug, route of administration, and length of treatment in the therapy of refractory OCD. In this protocol, subgroup analyses and sensitivity analyses were predefined. Clinical important and much-debated questions regarding differences in effects of lithium or various anticonvulsive drugs and length of treatment were investigated. Our results were important to clarify controversies and reduce uncertainty for both patients and healthcare providers. Implications for future research could be drawn from the results.

## Conflict of interest

The authors declare no conflict of interest.
